# Experimental hut resting and entrance behaviour of *Anopheles darlingi* from Zungarococha, a malaria endemic community in Loreto, Northern Peruvian Amazon

**DOI:** 10.1186/s12936-025-05442-2

**Published:** 2025-08-19

**Authors:** Fanny Castro-Llanos, Craig A. Stoops, John P. Grieco, Karin Escobedo-Vargas, Victor Lopez-Sifuentes, Audrey Lenhart, Frederick M. Stell, Ryan T. Larson, Nicole L. Achee, Gissella M. Vásquez

**Affiliations:** 1 U.S. Naval Medical Research Unit SOUTH, Bellavista, Callao, Peru; 2 Department of Biological Sciences, University of Notre Dame, Notre Dame, IN USA; 3 Division of Parasitic Diseases and Malaria, Entomology Branch, Centers for Disease Control and Prevention, Atlanta, GA USA; 4 Present Address: Naval Support Activity Hampton Roads, Defense Health Agency, Norfolk, VA USA; 5 Present Address: Citrus County Mosquito Control District, Lecanto, FL, USA; 6 Present Address: Navy and Marine Corps Force Health Protection Command, Portsmouth, VA USA

**Keywords:** *Anopheles darlingi*, Malaria, HLC, Experimental huts, Resting behavior, Entry behavior, Peruvian Amazon

## Abstract

**Background:**

*Anopheles darlingi* is a primary malaria vector in the Peruvian Amazon, yet characterization of behavioural traits contributing to human-vector contact is limited. Additionally, studies comparing key behaviours of wild-type to colonized *An. darlingi* populations are minimal. This study compared resting and entry behaviour between these two types of populations. Specific objectives were to use experimental huts to (1) evaluate and compare indoor resting behaviours of wild-type and colonized *An. darlingi* populations; (2) quantify *An. darlingi* house entry rates into interception traps in relation to protected Human Landing collection (HLC).

**Methods:**

The study was conducted in Zungarococha village, Loreto, Peru. Prior to hut evaluations, *An. darlingi* biting activity and population dynamics were evaluated using HLC outside local homes from June 2014 to May 2015. Indoor resting location (window, door, wall, roof) of wild caught and colonized *An. darlingi* was evaluated in three experimental huts. Controlled indoor releases were performed for 6 days each month from March to August 2015. Wild *An. darlingi* hourly house-entry rates were quantified using interception traps affixed to an experimental hut and indoor HLC from May to August 2015. Two collectors were positioned inside huts to generate host-seeking cues during evaluations.

**Results:**

*Anopheles darlingi* had a bimodal outdoor biting pattern with two peaks at 1800 h and 2200 h. HLC densities were associated with Amazon River levels measured nearby Iquitos city. Colonized *An. darlingi* preferred to rest in lower parts of the door (29.8%), roof (12.7%), and window (11.8%) which was similar to wild caught *An. darlingi* which preferred to rest in low door (32.7%), window (14.0%), and roof (13.0%). Wild *An. darlingi* entry behaviour peaked from 2200 to 2300 h, this was clearly observed when collection densities increased, varying between 2300 and 2400 h at low collection densities. Capture rates from interception traps were lower compared to indoor HLC rates from adjacent experimental huts and local houses.

**Conclusions:**

Results from this study provide useful insights into *An. darlingi* resting and entry behaviour in a malaria endemic area in the Peruvian Amazon and inform on the use of colonized mosquitos in vector behavioural studies. This information is relevant to malaria epidemiology and will be useful to evaluate new tools for malaria control programmes.

## Background

*Anopheles darlingi* is one of the most efficient malaria vector species in the Americas due to its vector competence and pronounced anthropophilic behaviour [1–7]. *Anopheles darlingi* was originally described in Venezuela, and has a large geographic distribution, extending from southern Mexico to Northern Argentina, including populations in Colombia, Brazil and Peru [8–10]. Although the number of reported cases dropped considerably in 2019, malaria continues to be an important public health problem in the Loreto region of the Peruvian Amazon, where *An. darlingi* is the most important malaria vector [11–15].

Understanding the ecology and behaviour of malaria vectors is essential in the search for new tools and strategies for malaria control [16, 17]. For example, knowing whether malaria transmission predominantly occurs inside or outside houses is important in the implementation of vector-centered malaria control programmes. Anti-vector interventions such as indoor residual spraying (IRS) or insecticide-treated nets (ITNs) are generally effective when human-vector contact occurs mainly indoors. Several researchers have described the ease with which *An. darlingi* enters houses to blood feed, which likely led to the high efficacy of malaria control campaigns with IRS using dichloro-diphenyl-trichloroethane (DDT) in the 1950s in Venezuela and Central America [18–20]. 

Experimental hut evaluations have long been used to characterize behavioural traits of malaria vectors in the Americas, Africa, and Asia [21–29]. The first mosquito experimental huts were built in Uganda during the 1940s to evaluate the efficiency of residual insecticides in malaria campaigns, and huts continue to be used in many countries to evaluate novel insecticides and repellents [30, 31]. Experimental huts are ideally built to mimic local houses with the benefit of having a controlled interior space compared to actual residences to facilitate standardization of variables, including the number of human occupants, number of entrance portals, and type of construction material [22, 25]. Experimental hut studies have been conducted for characterizing the behaviour and control of *An. darlingi*. Rozendaal [32], evaluated the biting patterns and efficacy of insecticide treated materials against *An. darlingi* in Suriname. Sachs et al. [23] evaluated and compared the entry and exit patterns of *An. darlingi* populations from Belize and Peru using interception traps affixed to experimental huts which reported differences between the two study locations. In Belize, *An. darlingi* exhibited a bimodal entry pattern occurring between 1900–2000 h and 0500–0600 h and peak exiting occurring between 1900 and 2000 h [23] while in Peru, *An. darlingi* demonstrated peak entry between 2200 and 2300 h and peak exiting between 2300 and 2400 h. Research has been conducted into whether or not such documented differences in *An. darlingi* behavioural patterns from across the species geographic range [19] is in fact a result of a potential subspecies genetic shift [33, 34]. Regardless, these demonstrative behavioural differences highlight the importance of characterizing local vector population traits that relate to the probability of human-vector contact (vector competence), thereby affecting malaria transmission, for developing more effective mosquito control interventions. There is increasing evidence that some malaria vector species may be shifting towards feeding outside versus inside [35–37], which may be occurring in the Loreto region, thus threatening indoor-focused malaria vector control campaigns [2, 19, 38, 39]. Recently, Saavedra and collaborators found a higher *Plasmodium* infection rate in *An. darlingi* mosquitoes collected outdoors than those collected indoors by HLC in communities around Loreto, confirming important *An. darlingi* exophagic behaviour [40]. 

There is evidence that the behaviour of laboratory populations of *Anopheles* could differ compared to a wild population. Lainhart et al*.* reported a possible genetic difference and observed a decrease in heterozygosity between laboratory and *An. darlingi* populations in the colonization process [41]. Birnberg et al*.* found a variation in the microbiota of *Anopheles atroparvus* captured in the field and subsequent generations of the same mosquito raised in the laboratory during the colonization process [42]. The present study used an *An. darlingi* colony that was established by the U.S. Naval Medical Research Unit South (NAMRU SOUTH) in 2013 from mosquitoes collected from Zungarococha village located in Iquitos, Peru [43] and compared resting behavioural patterns of wild population of *An. darlingi* from Zungarococha village to the colonized *An. darlingi.* A F_1_ generation of wild caught mosquitoes was also used and compared with *An. darlingi* from later generations. 

Continuous use of insecticide-treated nets (ITNs) and indoor residual spraying (IRS) can produce resistance to insecticides and modifications in mosquito behaviour that include changes in the biting and resting behaviours of malaria vectors [35, 44, 45]. During insecticide application studies in the 1960s, Mathis noted mosquito resting sites and recaptured more than 94% of *Anopheles gambiae* and *Anopheles funestus* resting on the ceiling [46]. Roberts et al*.* studied in Brazil the indoor-resting behaviour of *An. darlingi* females during and after feeding, observing 58.7% resting on the ceiling and 37% resting on the walls [47]. Rozendal et al*.* observed that *An. darlingi* females rested for an average of 4 h after blood feeding and that they did so very close to the entrance door of the experimental houses [48]. Quiñones et al*.* observed the tendency of *An. darlingi* from Colombia to rest below 1.5 m [49]. Paajimans et al. highlighted the importance of understanding mosquito resting behaviour and its association with the microclimate in both mosquitoes with exophilic behaviour and mosquitoes with endophilic behaviour [50].

Experimental huts are standardized constructions used to perform evaluations under controlled conditions (semi-field trials) and are often used to carry out evaluations of (LLINs), topical repellents or spatial repellents [51]. The experimental huts are generally designed to mimic local houses and can be equipped with entry and exit traps which allows the entry and repellency rates to be measured, respectively [52]. 

Since *An. darlingi* is an anthropophilic mosquito, the use of humans as an attractant is an effective collection method and allows for certain aspects of vector competence to be studied. Human landing catches (HLC) is the gold standard methodology for assessing malaria vector biting behaviour in anthropophilic Anophelines, such as *An. darlingi* [53, 54]. Alternative and comparable methods to HLC are a continued research focus [55, 56].

In this study, humans were employed as attractants inside experimental huts equipped with interception traps to measure the entry patterns of *An. darlingi*. Entry patterns were then related to the biting rate obtained from conducting HLCs inside experimental huts and local houses.

## Methods

### Study site

**Fig. 1:**
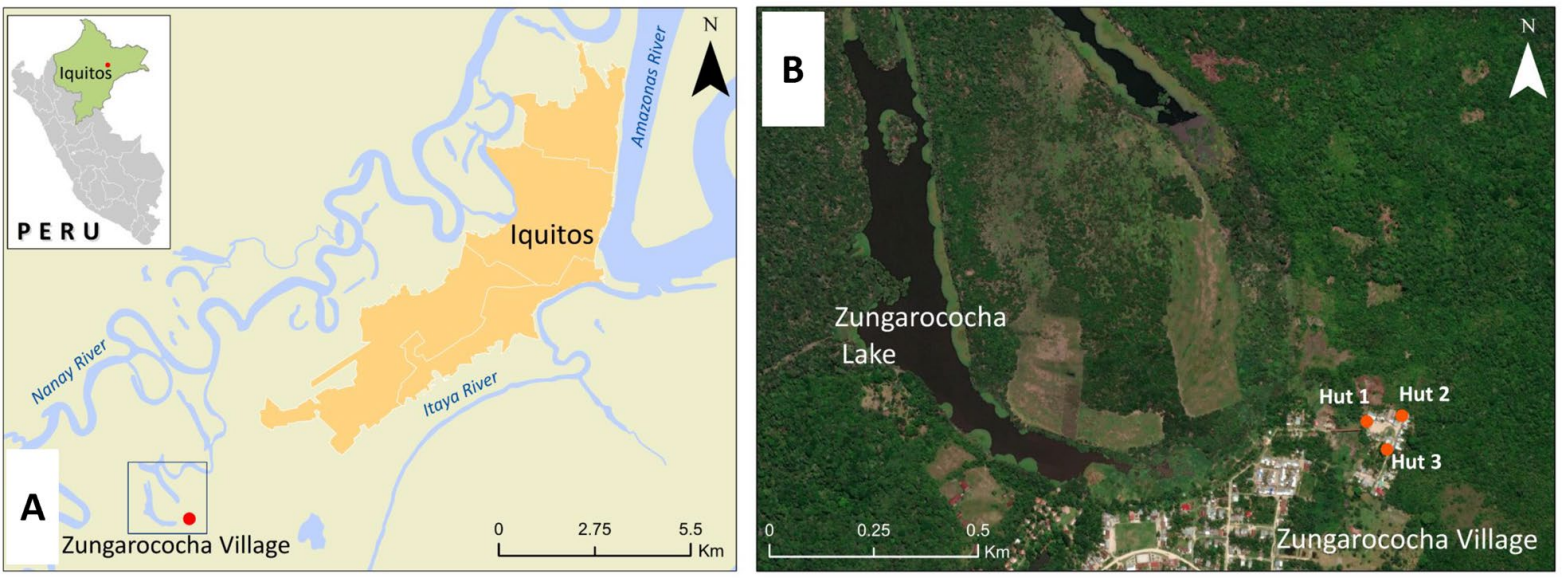
Study site: **A** Zungarococha village (green rectangle), a malaria endemic community in Loreto, Peru; **B** Experimental huts location within Zungarococha village (red circles)

The study was conducted in Zungarococha (3.8226 S, 73.3500 W), a malaria endemic community [57–59], located near the Nanay River, 18 km southwest of Iquitos, in the Loreto Region of Peru (Fig. 1). Experimental huts were positioned adjacent to local houses of the "Victor Mori" neighborhood, approximately 300 m from lake Zungarococha and around 50 m from the forest edge.

### Experimental huts

**Fig. 2:**
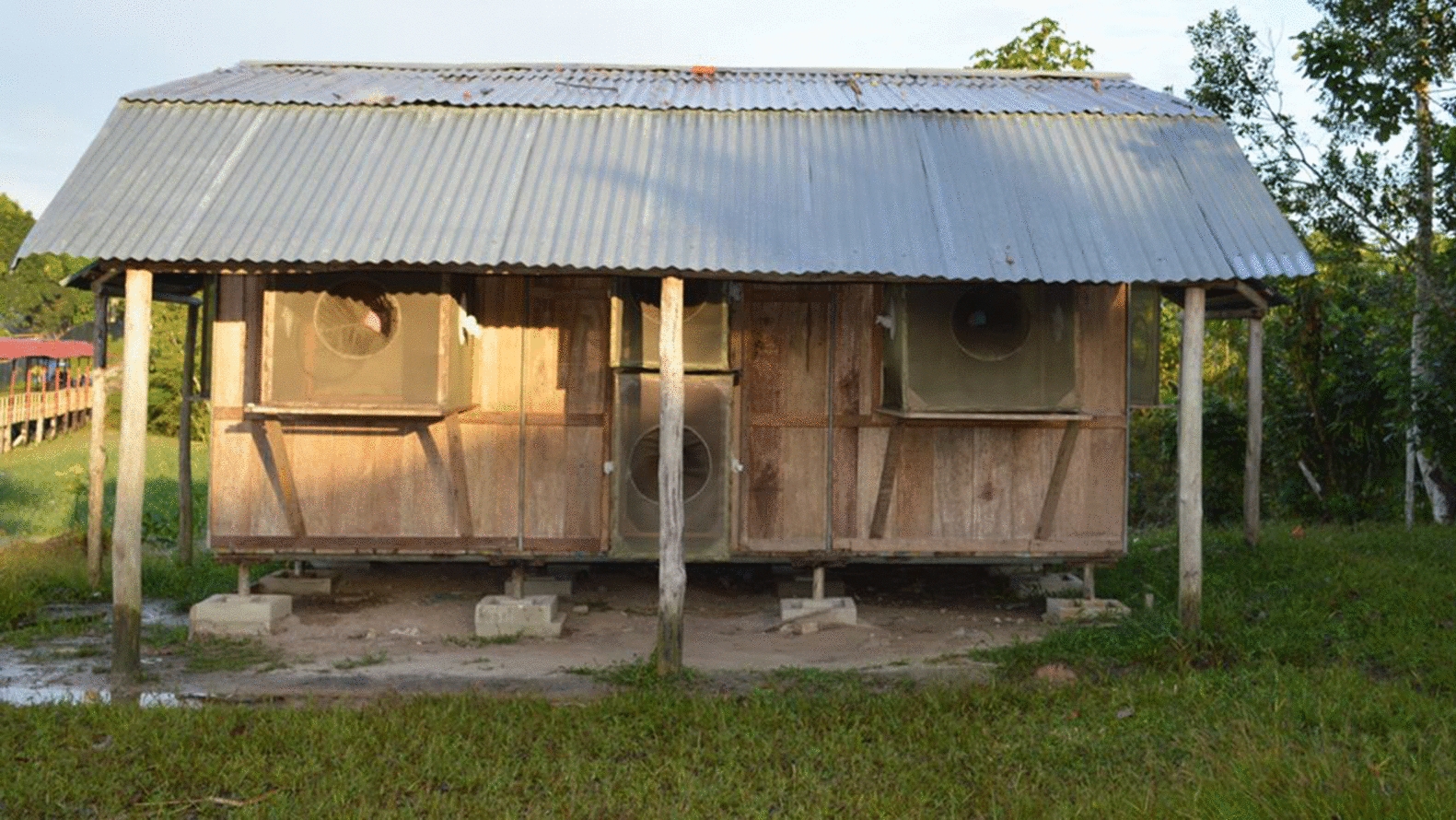
Experimental hut in Zungarococha village, Loreto, Peru

Huts were constructed to mimic local houses (Fig. 2) [23], and each hut (4 m x 5 m × 3.4 m) was raised 45 cm above ground level. Walls (2 m high) and floors were constructed using non-treated pieces of quillosisa wood with galvanized pipes used to frame the structures. A-frame roofs were made using corrugated zinc. The wood floors were covered with manufactured white plastic to facilitate observations of mosquito knockdown (KD) and mortality. Each hut had four windows (1 m x 1 m) with two windows located at the front of the structure and two at the sides; two doors, one (1 m × 2 m) located at the front and other auxiliary door (80 cm × 1 m) located at the side, and eave spaces (30 cm) between the wall and roof at all four sides of the hut. The four windows and the principal door were affixed with interception traps for collecting mosquitoes during entry experiments. Door traps (Fig. 2) were separated into upper and lower halves to facilitate removal of mosquitoes from the trap. Wooden-framed interception traps were lined with green polyester mesh attached to the frame, with two sleeves on the side of each trap to allow for mosquito removal. Eave gaps were covered with polyester netting to prevent mosquito passage. A green polyester curtain was used to cover the auxiliary door opening while collectors entered and left the huts.

### Biting activity and seasonal population dynamics

Prior to this study there was no information on the densities of *An. darlingi* in Zungarococha village that also considered seasonal changes. For this reason, monthly HLC collections were conducted outside local homes from June 2014 to May 2015 and prior to indoor resting and house entry evaluations. Personnel performing this activity were provided protective clothing including leather shoes, long pants rolled up below the knee, a long-sleeved shirt, a fine mesh jacket with hood and black long stockings to cover the exposed leg, as well as malaria chemoprophylaxis under NAMRU SOUTH Occupational Health surveillance [60]. All *Anopheles* were collected from 1800 to 0500 h (forty minutes of collection per person per hour) for a 3-night period each month. Collections were made throughout the month. Human landing rates (HLR) were calculated as the number of landings (bites) per person per night. According to the Peruvian Ministry of Health, *An. darlingi* constitutes 90% of the Anophelines in the peripheral zone of Iquitos in the rainy season and although the population decreases in the dry season, it continues to be the predominant Anopheline during dryer periods [17, 61, 62]. Reinbold-Wasson et al*.* observed the relationship between the level of the Amazon River and biting rates of *An. darlingi* at Puerto Almendra, a community located on the peripherical zone of Iquitos [63]. Relationships between the HLR and the Amazon River level were examined. Since water level data for many of the Amazon River tributaries is lacking, river level data were obtained from the Peruvian Navy Service of Hydrography and Navigation for the Amazon (SEHINAV) as a proxy for the Nanay River.

### Indoor resting behaviour evaluation

Three experimental huts were used simultaneously during indoor resting studies. Indoor resting behaviour of wild caught and colonized female *An. darlingi* was evaluated for six consecutive nights each month during a 6-month period from March to August of 2015. F_1_ generation mosquitoes were obtained from the collection of adult females in the study location using protected HLC, and the captured mosquitoes were provided with cotton wool moistened with 10% sucrose solution and were transported to the laboratory in Styrofoam boxes. In the laboratory, they were allowed to feed on chicken blood through an artificial feeding system with glass feeders, and after 2 days the wings were removed, and they were placed in 9-dram vials with lids with the necessary conditions so that they could lay eggs. Eggs were collected and transferred to breeding trays, and when the larvae turned into pupae, they were placed in cages to develop into adults. Wild-caught *An. darlingi* populations used in these studies were collected using HLCs the night before a trial. Populations of colonized and first generation (F_1_) *An. darlingi* female mosquitoes (3–7 day old) were reared at NAMRU SOUTH Entomology laboratory in Iquitos using established NAMRU SOUTH protocols as described by Villarreal et al*.* [43] were 24 h sugar-starved.

**Fig. 3:**
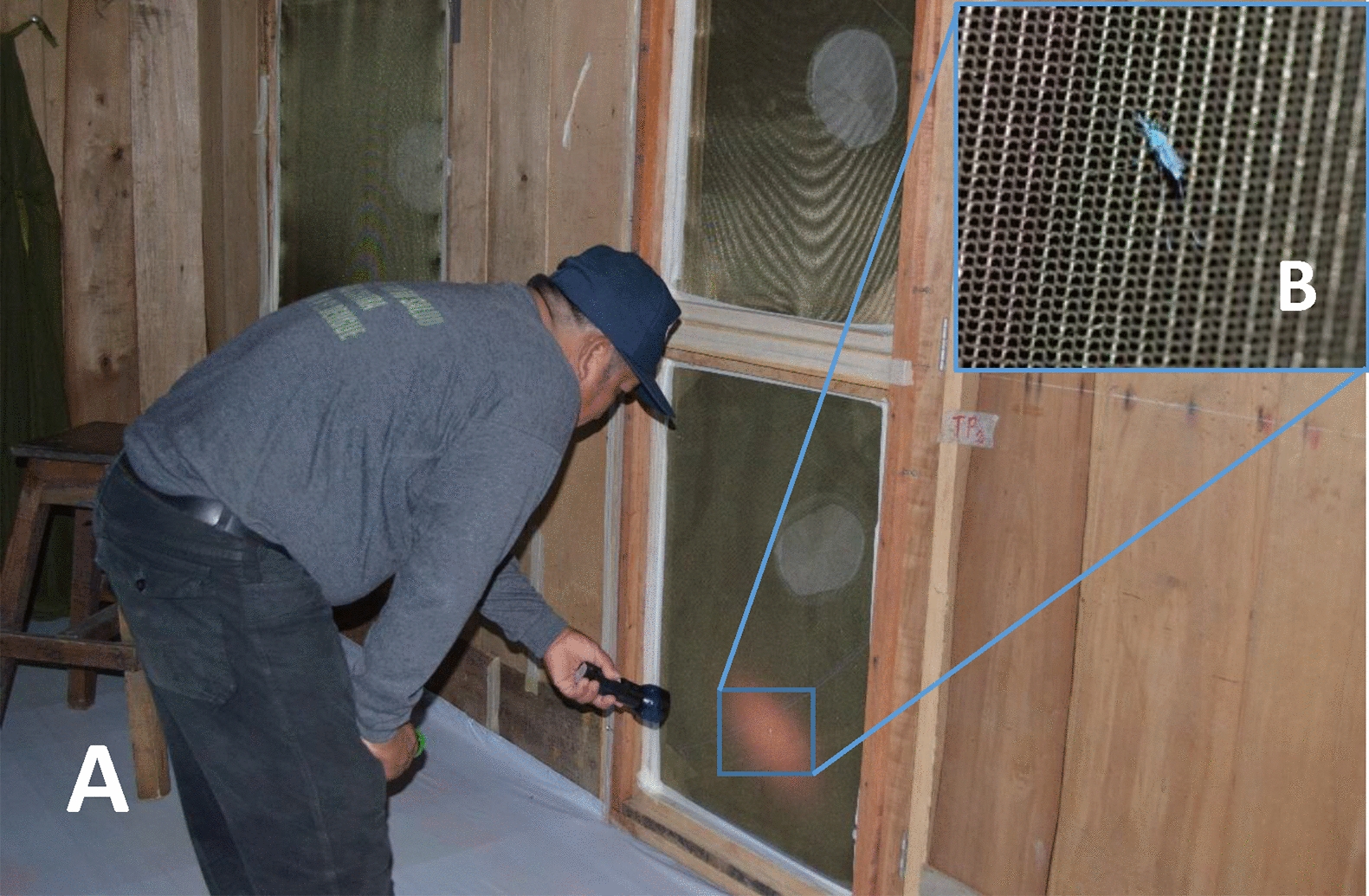
**A** *Anopheles darlingi* indoor resting evaluation; **B** *An. darlingi* dusted with fluorescent powder resting on the lower section of the door

A total of 1,404 h of resting evaluations were performed: eighteen trials (702 h of evaluation) using wild-caught vs. colonized (F_27_ to F_29_) *An. darlingi*, and eighteen trials (702 h of evaluation) using F_1_ vs. colonized (F_26_, F_30_, and F_31_) *An. darlingi* (Table 1). Six hours prior to each experiment, 25 females per test cohort were marked with fluorescent powder (BioQuip Products, Inc., Gardena CA) (Table 1) (Fig. 3B) by brushing a loaded paintbrush against the mesh net covering the cardboard cup containing the mosquitoes. Each mosquito cohort was marked with a unique dye color which was rotated during the experiments to facilitate calculating the proportion of *An. darlingi* that rested per day and per release group as there was the possibility of finding mosquitoes released the day before. After marking, females were transferred into a clean 0.9 L plastic container. A water-moistened cotton pad was placed on top of the containers, and test populations were kept in a humidified cooler until the start of the experiment.

**Table 1:**
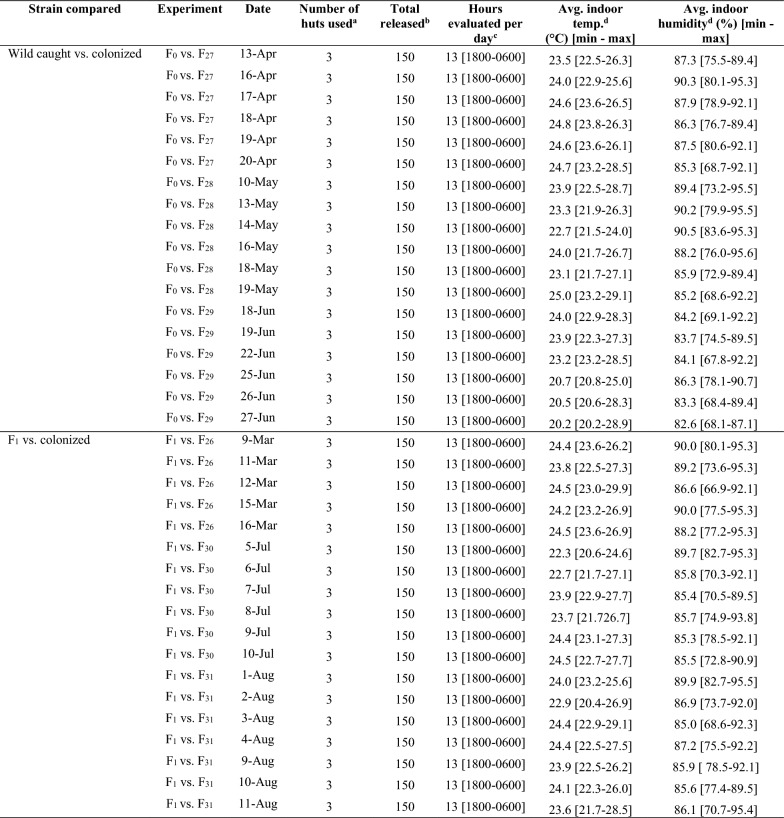
Description of *Anopheles darlingi* resting strain comparisons between wild-caught versus colonized populations (F_27-29_) and between F1 mosquitoes versus colonized populations (F_26-31_) from Zungarococha, Loreto, Peru

^a^Three experimental huts were used for each day of the experiment

^b^Twenty-five mosquitoes of each strain were released in each experimental hut for each day of the experiment

^c^In total 13-h × 18 days × 3 huts = 702-h of observations in each block of experiments

^d^Average of 12-h of evaluation

**Table 2:** *Anopheles darlingi* resting observations for wild-caught (F_0_) or F_1_ mosquitoes versus colonized populations (F_26-31_) from Zungarococha, Loreto, Peru

Number of observations	Wild caught vs. colonized							F_1_ vs. colonized					
	Mean ± SE hourly % Resting^a^			Mean ± SE hourly % KD				Mean ± SE hourly % Resting^a^			Mean ± SE hourly % KD		
	F_0_	F_27-29_	*P*^b^	F_0_	F_27-29_	*P*^b^		F_1_	F_26-31_	*P*^b^	F_1_	F_26-31_	*P*^b^
702	42.3 ± 0.8	22.7 ± 0.6	< 0.0001	3.0 ± 0.2	3.9 ± 0.2	< 0.0001		31.5 ± 0.6	27.8 ± 0.6	< 0.0001	5.5 ± 0.2	5.5 ± 0.2	0.8232

^a^Percent resting after correcting for knock down (KD) inside the hut

^b^P-value for Wilcoxon rank-sum test (α = 0.005)

Marked populations were released inside of designated huts at 1730 h for all trials. Resting location was recorded by study technicians using a UV flashlight (black light, 51 LED), for ten minutes each hour starting at 1800 h and continuing at the top of each hour until 0600 h (Fig. 3A). Field technicians rotated between experimental huts each sampling day to reduce observer bias. For each observation, resting locations were recorded as: floor, door, wall, window, ceiling, eave, auxiliary door curtain or ‘other’. The number of observed marked mosquitoes resting on the higher (> 1 m) and lower (< 1 m) parts of the doors and walls was also recorded. To assess knock down (KD), the number of fallen mosquitoes on the floor was counted every hour. In these release and recapture experiments, KD was used to correct the number of available marked mosquitoes to observe. After 12 h of observation, mosquitoes within the huts were recaptured using both manual and Prokopack aspirators (John W. Hock Company) for an average of 15 min. Before starting a new day of mosquito collections, each hut was cleaned with soap and water to keep away ants and spiders.

Temperature and relative humidity (RH) were recorded inside each hut using HOBO^®^ data loggers (H08-004-02, ONSET Computer Corporation, Massachusetts). External parameters including temperature, RH and rainfall were recorded using an ONSET Computer Corporation weather station data logger (H21-001).

### Entry behaviour evaluation

**Fig. 4:**
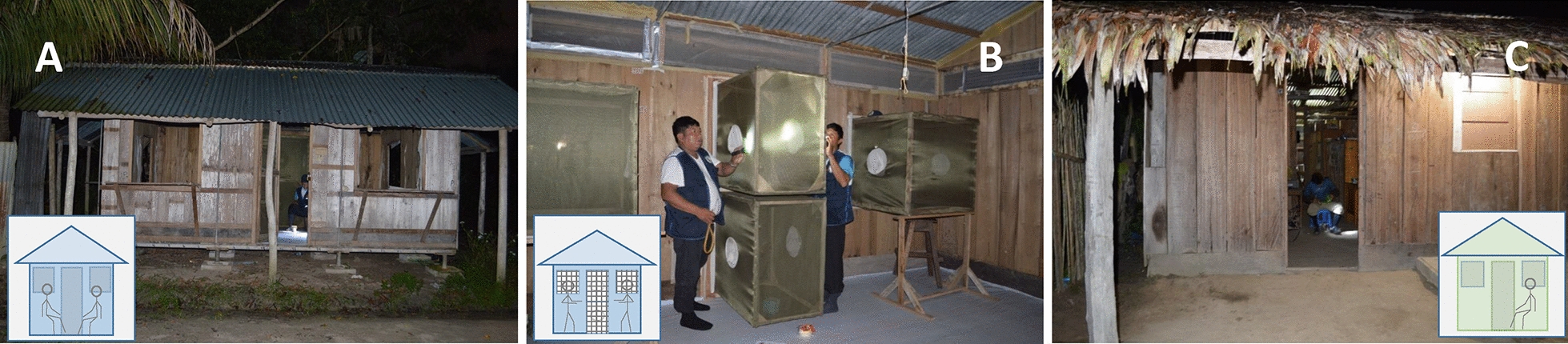
House Entry Evaluation: **A** Protected human landing catches (HLC) performed inside the experimental hut; **B** Collection of mosquitoes from entry traps inside experimental hut; **C** HLC performed

**Fig. 5:**
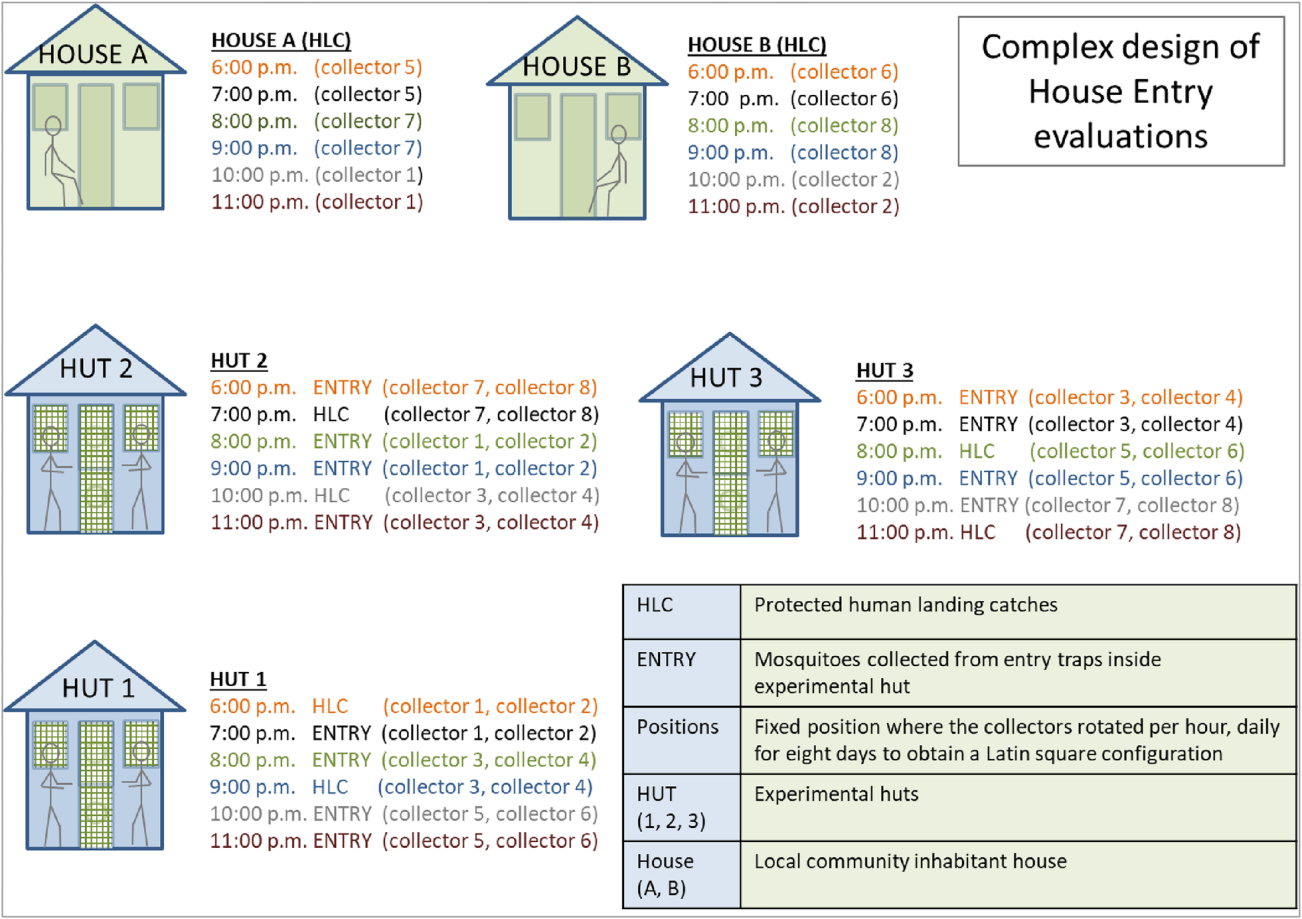
Experimental design used for *An. darlingi* house entry evaluations: eight collectors rotated per hour, per hut or house and per day. The figure is the pic of the first day of the trial that rotated across time (eight days of collection)

Three experimental huts without traps (Fig. 4A) or affixed with four window and two door interception traps (Fig. 4B) and two local houses without traps (Fig. 4C) were used for evaluating *An. darlingi* entry behaviour patterns. These experiments were set up so that the same experimental huts were used with and without interception traps; the technical staff was trained to place and dismantle the traps in less than 10 min for this purpose. Experiments were performed during three separate 8-day blocks from May 2015 to August 2015 (Table 4). Preliminary experiments showed that the location of the experimental huts and conditions in the local houses could affect the number of mosquitoes collected. In addition, the individuals conducting the HLCs exhibited varying levels of attractiveness to mosquitoes. During each block of experiments the three experimental huts and the two local houses were used simultaneously based on a complex Latin square experimental design, where eight trained collectors rotated per hour, per hut/house and per day, during the eight consecutive collection days (Fig. 5). Due to the number of people required for this experimental design, only two local houses could be evaluated. Two collectors performed HLCs inside experimental huts because preliminary experiments showed that a single collector did not generate enough host cues to attract *An. darlingi*. Only one collector performed HLC in the local houses because inhabitants were also present in the houses. The collections were for six consecutive hours, from 1800 to 2300 h. Two collectors were positioned inside each experimental hut and local house to generate host-seeking cues 30 min before the start each trial at 1730 h. Inside experimental huts, HLCs were conducted for 30 min each hour from 1800 to 2300 h by two collectors separated from each other by a meter of distance (Fig. 4A). Simultaneously, inside the local houses, HLCs were conducted by a single collector for the same period of time (Fig. 4C).

Inside experimental huts with interception traps, two collectors set the traps and then positioned themselves inside to generate host-seeking cues 30 min before trap collection began. Once collections had commenced, the two collectors aspirated mosquitoes from interception traps (3 min/trap) from 1830 to 2330 h (Fig. 4B), based on previous research [23, 64]. Collected mosquitoes were placed in cardboard cups and transported to the field laboratory in Zungarococha, where they were identified using morphological keys by Faran and Linthicum [8] and Consoli and Oliveira [65]. Fifty percent of the *An. darlingi* females collected were dissected to determine reproductive status [66, 67]. Temperature and relative humidity were recorded inside each experimental hut and local house. Outside temperature, relative humidity and rainfall were recorded using a weather station data logger.

### Data analysis

Analyses were performed using the statistical package STATA 16.1, (StataCorp, TX, USA).

### Biting activity and seasonal dynamics

*Anopheles darlingi* collected per person per hour was averaged to identify the activity patterns of this mosquito at Zungarococha village during a 12-h nightly period. Because the collections were for 40 min, the number of mosquitoes collected was multiplied by 1.5 to estimate the number collected per hour. The HLR was graphed by month to observe the population dynamics through the 12 months studied. Pearson's correlation was performed to investigate the association between HLR and the monthly level of the Amazon River.

### Indoor resting

Resting activity was evaluated using the hourly percent resting corrected by the KD number. Percent resting for wild vs. colony populations and F1 vs. colony populations was averaged per hour for each set of tests to identify resting patterns. The comparison of resting activity between populations was performed for each set of experiments using the nonparametric Wilcoxon rank-sum test. The hourly percent of KD was also evaluated for each set of experiments using the same test. The preference for resting location (high wall, low wall, high door, low door, window, eave, roof, curtain, floor and others) was evaluated with data from each set of experiments, (wild vs. colony populations) and (F1 vs. colony populations), through multiple comparisons between each pair of resting places using Kruskal–Wallis (Wilcoxon rank test). Pearson's correlation was performed to investigate the association between resting behaviour and three environmental parameters: temperature, relative humidity, and rainfall amount.

### House entry evaluation

The total number of *An. darlingi* females captured per hour was averaged to identify behaviour patterns by treatment (entry traps, HLCs in experimental huts and HLCs in local houses). The number of events observed per treatment was obtained by Long-rank test for equality of survivor functions. Entry behaviour patterns were compared between treatments by performing an analysis of variance for repeated measures of pair-wise comparisons between treatments. The temperature and relative humidity recorded inside huts and houses were compared every two treatments using Kruskal–Wallis (Wilcoxon rank test). Pearson's correlation was used to examine the association between entry behaviour and three environmental parameters. Reproductive physiological status of *An. darlingi* collected and dissected from house entry evaluations was analyzed using Chi-squared tests to identify treatment effects (entry traps, HLC in experimental huts and HLC in local houses).

## Results

### Biting activity and seasonal dynamics

**Fig. 6:**
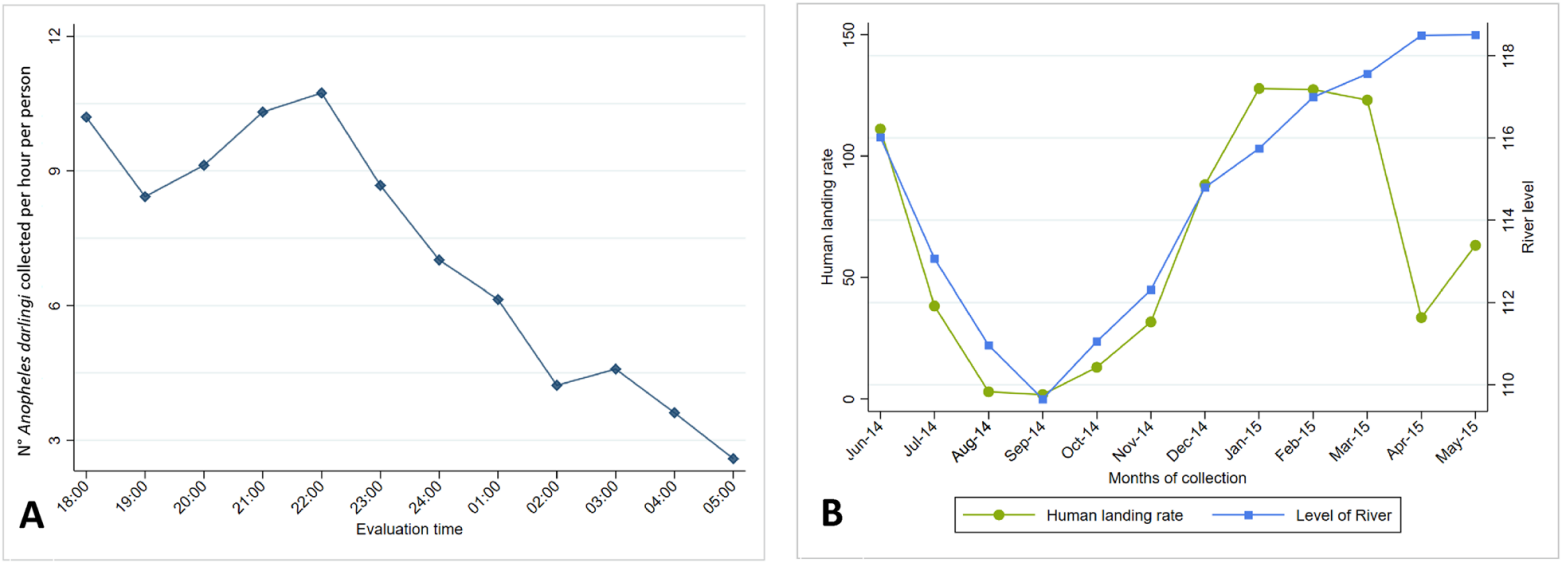
Activity of *Anopheles darlingi* at Zungarococha village, from June of 2014 to May of 2015; **A** biting activity of *An. darlingi* at night per 12-h period (1800 h to 05:00 h); **B** dynamic of population: human landing rates (number of *An. darlingi* captured outside houses per 12-h period (1800 h to 0500 h) per person in relation with river level

A peak in HLC at our study site showed a bimodal pattern with a first peak at dusk 1800 h and a second peak before midnight at 2200 h (Fig. 6A). *Anopheles darlingi* densities, measured at HLR, ranged from 1.8 to 128 landings per person per 12-h period (1800–0500 h). These were the highest in June 2014, and decreased once the dry season began (after June), reaching their lowest level in September 2014, and increased again considerably when the rainy season resumed in December 2014 (Fig. 6B). The HLR was positively associated with river water levels (*r* = 0.6901, *P* = 0.013, Pearson’s correlation).

### Indoor resting

**Fig. 7:**
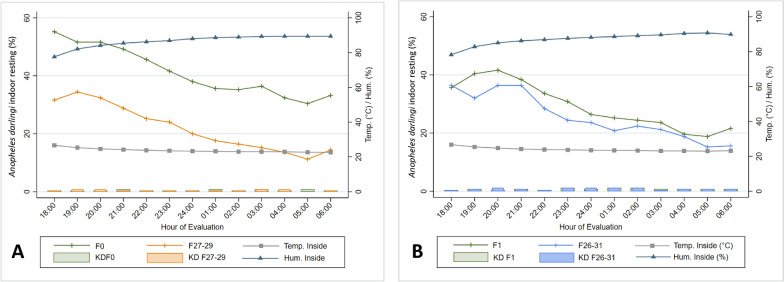
*Anopheles darlingi* percent indoor resting and knock down recorded hourly from 1800 to 0600 h, and environmental parameters (T°, RH%) recorded in experimental huts; **A** wild caught (F0) and colonized populations (F27–F29); **B** F1 and colonized populations (F26–F31). The scales of both charts are different in order to visualize the trend

The peak of indoor resting for wild caught, F_1_ and colonized *An. darlingi* occurred between 1800 and 2100 h (Fig. 7A, B). Although patterns were similar among populations, a higher percentage of wild caught and F_1_ *An. darlingi* were found resting indoors relative to colonized mosquitoes (Fig. 7A, B).

In the first experiment, the median percentage of resting *An. darlingi* was 42.3 and 22.7 in wild and colonized populations, respectively. There was strong evidence that a higher proportion of wild mosquitoes rested indoors in comparison to colonized mosquitoes (*P* < 0.001, two-sample Wilcoxon rank-sum test). There was a higher proportion of KD in colonized mosquitoes in comparison to wild mosquitoes (*P* < 0.001, two-sample Wilcoxon rank-sum test) (Table 2). In the second set of trials, in which F_1_ mosquitoes were used instead of wild caught, the median resting percentage was 31.5 and 27.8 in F_1_ and colonized populations, respectively. In addition, F_1_ mosquitoes were more likely to rest indoors (*P* < 0.001, two-sample Wilcoxon rank-sum test) (Table 2).

**Fig. 8:**
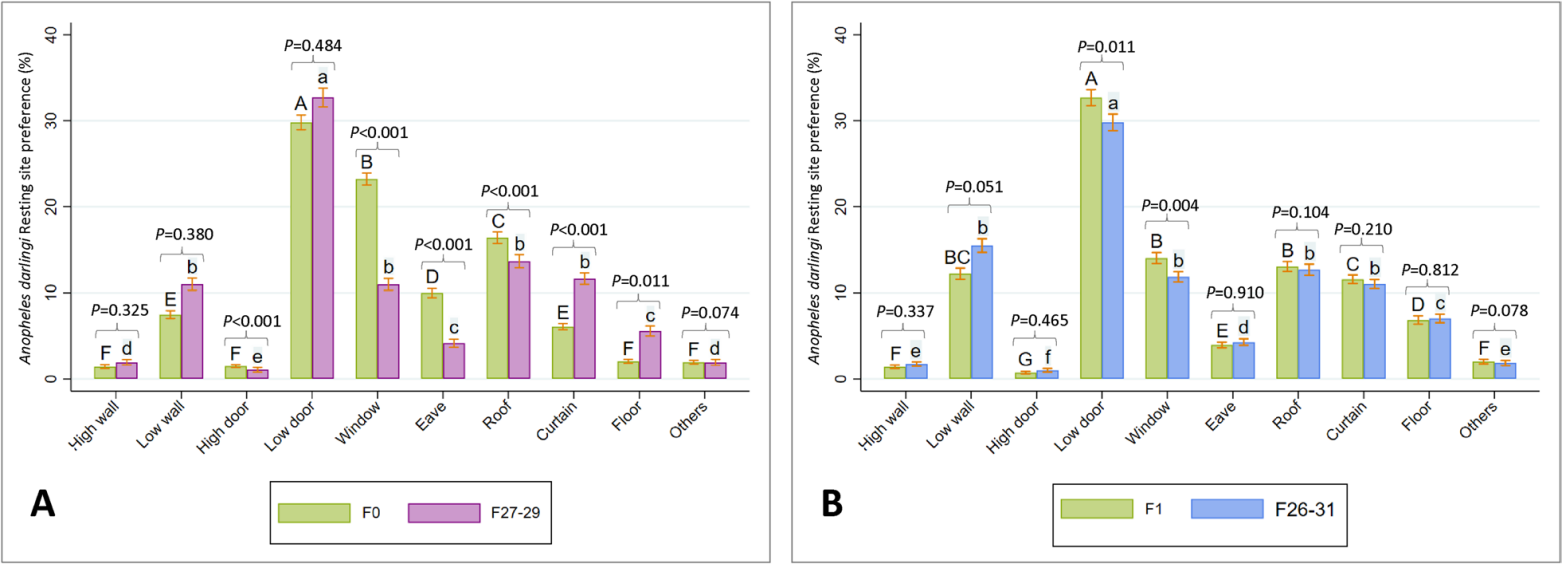
*Anopheles darlingi* indoor resting preference in experimental huts; **A** Bar graph created using average percent resting. **A** wild caught (F0) and colonized populations (F27–F29); **B** F1 and colonized populations (F26–F31). Different letters indicate significant differences based on Kruskal–Wallis equality-of-populations rank test

Percent resting for wild vs. colony populations: Wild caught *An. darlingi* preferred resting on the lower section of the door (29.8%), which was comparable with colonized mosquitoes (32.7%) (*P* = 0.484, Kruskal–Wallis equality-of-populations rank test). The second most common resting site for wild *An. darlingi* was the window (23.2%), while the second most common locations of colonized *An. darlingi* were the roof (13.6%), window (11.0%), the lower part of the wall (11.0%) and curtain (11.6%); this did not differ statistically (pair comparison using Kruskal–Wallis equality-of-populations rank test). The third through fifth most common locations for wild *An. darlingi* were the roof (16.4%), the eave (10.0%), and the curtain (6.1%), respectively; the high part of the wall (1.4%), the high part of the door (1.5%), the floor (2.0%) and other places (2.0%) were equally the sixth most common resting sites (pair comparison using Kruskal–Wallis equality-of-populations rank test). The third mostly commonly used locations for colonized *An. darlingi* was indistinctly the floor (5.6%) and eave (4.2%) (Kruskal–Wallis equality-of-population rank test). The fourth place of preference for colonized *An. darlingi* was indistinctly the high part of the wall (1.9%) and other places (1.9%) (Kruskal–Wallis equality-of-population rank test). The fifth and last place of preference of colonized *An. darlingi* was the high part of the door (1.1%) (Fig. 8A).

F_1_ vs. colony populations: The *An. darlingi* F1 generation: mosquitoes preferred resting in greater percentage on the lower section of the door (32.7%), it was similar in terms of preference but was not comparable with colonized mosquitoes (29.8%) (*P* = 0.011, Kruskal–Wallis equality-of-populations rank test). The second place of preference for *An. darlingi* F_1_ generation was indistinctly the window (14.0%), the roof (13.0%) and the low part of the wall (12.2%) (pair comparison using Kruskal–Wallis equality-0f-population rank test) while colonized *An. darlingi* indistinctly used the low part of the wall (15.5%), the roof (12.7%), the window (11.8%) and the curtain (11.0%) as the second preferred resting spot (pair comparison using Kruskal–Wallis equality-of-populations rank test). The third place of resting preference for *An. darlingi* F_1_ generation was the curtain (11.6%), while the third place of resting preference for colonized *An. darlingi* was the floor (7.0%). The fourth place of resting preference for *An. darlingi* F_1_ generation was the floor (6.9%) while the fourth place of resting preference for colonized *An. darlingi* was the eave (4.3%). The fifth place of resting preference for *An. darlingi* F_1_ generation was the eave (3.9%), while the fifth place of resting preference for colonized *An. darlingi* was indistinctly other places (1.9%) and the high part of the wall (1,7%) (*P* = 0.7410, Kruskal–Wallis equality-of-populations rank test); the sixth place of resting preference for *An. darlingi* F_1_ generation was indistinctly other places (2.0%) and the high part of the wall (1.4%) (*P* = 0.0164, Kruskal–Wallis equality-of-populations rank test) while the sixth place of resting preference for colonized *An. darlingi* was the high part of the door (5.6%) (Fig. 8B).

The hourly percentage of *An. darlingi* resting was positively associated with indoor temperature (*r* = 0.220, *P* < 0.001, Pearson’s correlation), but negatively associated with indoor humidity (*r* = −0.3454, *P* < 0.001, Pearson’s correlation). The hourly percentage of *An. darlingi* collected and the outdoor temperature showed a positive association (*r* = 0.1872,* P* < 0.001, Pearson’s correlation). In contrast, a lower percentage of *An. darlingi* were collected as outdoor humidity increased (*r* = −0.1222, *P* < 0.001, Pearson’s correlation). Rainfall did not significantly affect the percentage indoor resting of *An. darlingi* (*r* = −0.0295, *P* = 0.135, Pearson’s correlation).

### House entry evaluation

A total of 3,182 female *Anopheles* were collected from May to August 2015, of which 3,179 (99.9%) were *An. darlingi*. A total of 1,477 were collected by HLCs inside experimental huts (5.1 mosquitoes/person/hour), 1,071 were collected by HLCs inside local houses (3.7 mosquitoes/person/hour), and 631 were collected using experimental hut interception traps (2.2 mosquitoes/hour) (Table 3).

**Table 3:** Comparison between entry traps in experimental huts, protected human landing collection (HLC) inside experimental huts and inside local houses for *Anopheles darlingi* collected in Zungarococha, Loreto, Peru

Number of observations	Treatment	*Anopheles darlingi* collected	Mean ± SE hourly entry	Events observed^a^	Entry trap vs. Experimental Hut HLC	Entry trap vs. House HLC	Experimental hut HLC vs. House HLC
					*P*^b^	*P*^b^	*P*^b^
288	Experimental Hut entry trap	631	2.2 ± 0.2	182	< 0.0001	< 0.0001	< 0.0001
288	Experimental hut indoor HLC	1477	5.1 ± 0.4	246			
288	House indoor HLC	1071	3.7 ± 0.2	227			

^a^Events observed obtained by Log-rank test for equality of survivor functions

^b^P-value obtained by Analysis of variance for repeated measures (α = 0.005)

**Fig. 9:**
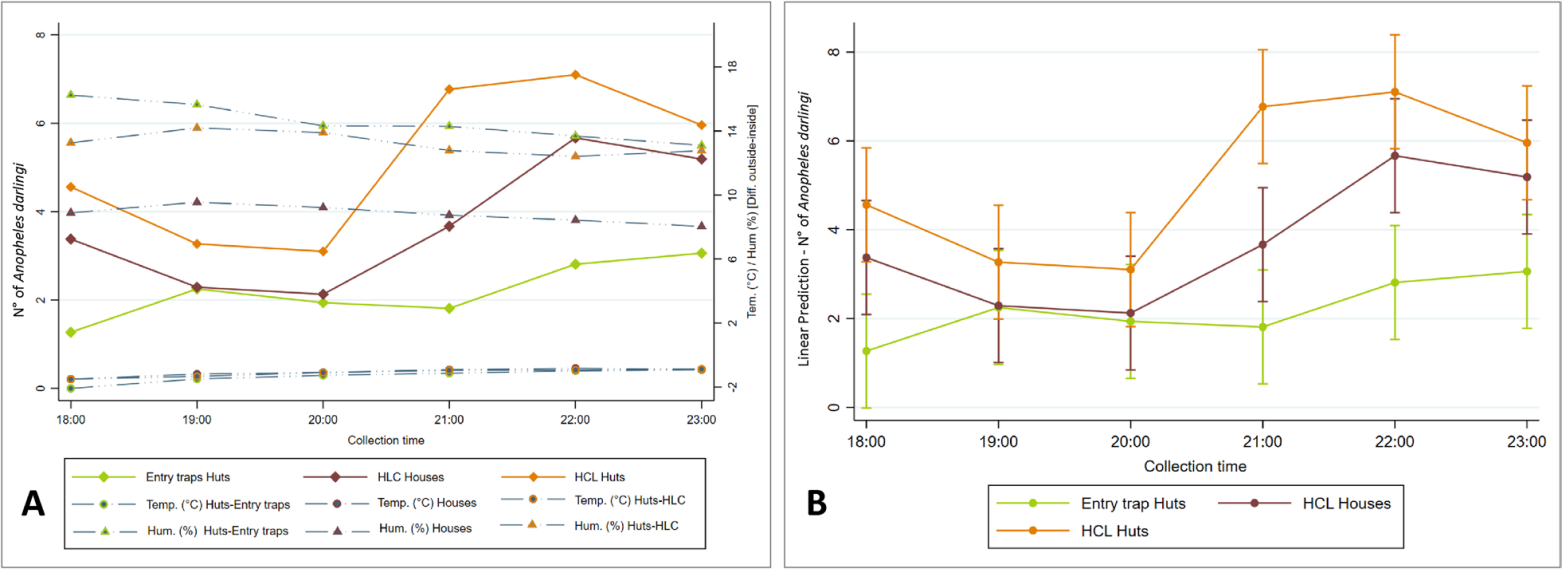
Average number of *Anopheles darlingi* collected in entry traps compared with human landing catch (HLC) inside experimental huts and local houses; **A** compared with environmental parameters recorded Temp (°C) and RH% [Difference outside-inside]; **B** Adjusted predictions for each time of collection [95% CIs]

Entry patterns among the three treatments (entry traps, HLC in experimental huts and HLC in local houses) showed that entry peaked between 2100 and 2200 h (Fig. 9A). This may vary according to the collection period. In the first block of experiments during May and June of 2015 a total of 1,504 *An. darlingi* was collected. During this period the collection peak was between 2200 and 2300 h, with 10.7 mosquitoes/person/hour inside local houses and 9.5 mosquitoes/person/hour inside experimental huts. Meanwhile, in the interception traps inside experimental huts, there was no noticeable peak for this type of collection (Table 4). During the second block of experiments in June and July a total of 632 *An. darlingi* was collected. There was a clear decrease in the number of mosquitoes collected; during this second period, with a collection peak inside local homes between 2200 and 2300 h (3.6 mosquitoes/person/hour), and a collection peak inside experimental huts between 2300 and 2400 h (3.5 mosquitoes/person/hour). For the interception traps, there was no noticeable peak of collection (Table 4). In the third period of collection during July and August of 2015 a total of 1,043 *An. darlingi* was collected (Table 4). In this period the collection peak inside experimental huts was between 2200 and 2300 h (8.2 mosquitoes/person/hour), the peak of collection inside interception traps was between 2300 and 2400 h (4.0 mosquitoes/hour), and there was no noticeable peak of collection inside local houses (Table 4).

**Table 4:** Average number of *Anopheles darlingi* collected in entry traps compared with protected human landing catch (HLC) inside experimental huts and local houses per block (Period of experiments) and hour of collection

Block period of eight experiments	Hour of collection	*An. darlingi* total collected	*An. darlingi* Entry traps in exp. huts Total (Mean ± SE)	*An. darlingi* HLC inside exp. huts Total (Mean ± SE)	*An. darlingi* HLC inside houses Total (Mean ± SE)
Block 1 [27-May to 15-Jun]	18:00	183	10 [0.6 ± 0.2]	85 [5.3 ± 1.0]	88 [4.9 ± 1.4]
	19:00	111	29 [1.8 ± 0.5]	30 [1.9 ± 0.6]	52 [2.9 ± 0.7]
	20:00	165	22 [1.4 ± 0.5]	82 [5.1 ± 1.4]	61 [3.4 ± 0.5]
	21:00	290	30 [1.9 ± 0.6]	144 [9.0 ± 1.6]	116 [6.4 ± 1.2]
	22:00	399	58 [3.6 ± 1.3]	152 [9.5 ± 2.3]	189 [10.5 ± 1.2]
	23:00	356	61 [3.8 ± 1.2]	134 [8.4 ± 2.4]	161 [8.9 ± 1.3]
Block 2 [30-Jun to 15-Jul]	18:00	94	11 [0.7 ± 0.3]	44 [3.1 ± 0.6]	39 [2.4 ± 0.6]
	19:00	109	26 [1.6 ± 0.6]	34 [2.4 ± 0.5]	49 [3.1 ± 0.9]
	20:00	78	19 [1.2 ± 0.3]	26 [1.9 ± 0.5]	33 [2.1 ± 0.5]
	21:00	109	24 [1.5 ± 0.6]	32 [2.3 ± 0.7]	53 [3.3 ± 0.7]
	22:00	121	19 [1.2 ± 0.4]	44 [3.1 ± 0.8]	58 [3.6 ± 0.1]
	23:00	121	22 [1.4 ± 0.5]	49 [3.5 ± 0.9]	50 [3.1 ± 0.6]
Block 3 [24-July to 14-Aug]	18:00	165	40 [2.5 ± 0.9]	95 [5.9 ± 1.1]	30 [1.9 ± 0.6]
	19:00	155	53 [3.3 ± 0.9]	78 [4.9 ± 1.6]	24 [1.5 ± 0.4]
	20:00	101	52 [3.3 ± 0.8]	34 [2.1 ± 0.6]	15 [0.9 ± 0.3]
	21:00	189	33 [2.1 ± 0.7]	128 [8.0 ± 2.0]	28 [1.8 ± 0.5]
	22:00	228	58 [3.6 ± 0.8]	131 [8.2 ± 2.0]	39 [2.4 ± 0.6]
	23:00	205	64 [4.0 ± 0.9]	102 [6.4 ± 2.3]	39 [2.4 ± 0.7]

For the entire period of experiments, the HLCs inside experimental huts showed 246 events observed, HLCs inside local homes showed 227 events observed, while the entry traps showed only 182 events observed (Table 3). No significant difference between the number of events of HLCs inside experimental huts and inside local houses (*P* = 0.0109, log-rank test for equality of survivor functions) was found, while a significant difference between the number of events in entry traps with the events of HLCs inside experimental huts (*P* < 0.001) and between HLCs inside local houses (*P* < 0.001, long-rank test for equality of survivor functions) was found. There is strong evidence that the hourly catches from HLCs inside huts versus entry traps differed (*P* < 0.001, analysis of variance for repeated measures), with 5.1 mosquitoes/person collected via HLCs inside huts and 2.2 mosquitoes collected in the interception traps of the huts. In addition, there is strong evidence on that the hourly HLCs inside local houses versus entry traps differed (*P* < 0.001, analysis of variance for repeated measures), with 3.7 mosquitoes/person collected via HLCs inside local houses. Additionally, more mosquitoes were collected in hourly HLCs inside huts in comparison to HLCs inside local houses (*P* < 0.001, analysis of variance for repeated measures) (Table 3). According to the plot of adjusted predictions, only one interaction was observed between HLCs in local houses and the collection with interception traps at 1900–2000 h; the lack of any other interactions supports our finding that the three types of collection are significantly different (Fig. 9B). The mean temperatures inside experimental huts during the collection with interception traps and without interception traps during HLCs were 25.6 ± 0.1 °C, and 25.4 ± 0.1 °C, respectively, and inside local houses was 25.3 ± 0.2 °C. We found no evidence that temperatures between these locations differed (all *P* > 0.05 using two-sample Wilcoxon rank-sum test). The mean relative humidity inside experimental huts during the collection with interception traps and without interception traps during HLCs were 81.7 ± 0.3%, and 83.0 ± 0.3 °C, respectively, and inside local houses was 87.3 ± 0.4 °C. There was no evidence that relative humidity between these locations differed significantly (all *P* > 0.05 using two-sample Wilcoxon rank-sum test).

*Anopheles darlingi* hourly entry showed no association with outdoor temperature (r = −0.0292, *P* = 0.3908), but did show an association with indoor temperature (r = −0.0809, *P* = 0.0319, Pearson’s correlation), and with the difference of temperature [outside-inside] (r = 0.0915, *P* = 0.0151). There was strong evidence that hourly entry was positively associated with indoor humidity (r = 0.2583, *P* < 0.001, Pearson’s correlation) and with outdoor humidity (r = 0.1683, *P* < 0.001, Pearson’s correlation). There was strong evidence that hourly entry was negatively associated with the difference of relative humidity [outside-inside] (r = −0.2147, *P* < 0.001, Pearson’s correlation). The average *An. darlingi* collected in entry traps compared with HLCs inside huts and local houses was plotted in comparison with the difference [outside-inside] of temperature (°C) and relative % humidity (Fig. 9A). *Anopheles darlingi* hourly entry and rainfall showed no association (r = −0.0389, *P* = 0.2348, Pearson’s correlation).

The ovaries of 1,552 *An. darlingi* (48.8% of total collected) were examined to determine reproductive status. There was no evidence that the rates of nulliparous, parous or gravid females differed between those collected in entry traps, HLCs in experimental huts and HLCs in local houses (*χ*2 = 5.5803, *P* = 0.233) (Table 5). Of the mosquitoes dissected, 64.7–68.6% were in the parous stage, 24.5% to 30.7% in the nulliparous stage, and 3.1–4.3% in the gravid stage (Table 5). However, the rates of nulliparous, parous or gravid females differed between blocks of experiments (temporal variation) (*χ*^2^ = 71.8107, *P* < 0.001) (Table 5). In the first block of experiments between May and June the percentage of nulliparous was 37.3%, the percentage of parous was 59.9%, and the percentage of gravid was 2.7%; in the second block of experiments between June and July the percentage of nulliparous was 31.9%, the percentage of parous was 63.3% and the percentage of gravid was 4.9%. In the third block of experiments the percentage of nulliparous decreased to 15.0% and the percentage of parous increased to 80.8%, while the percentage of gravid was 4.2% (Table 5).

**Table 5:** Reproductive physiological status of *Anopheles darlingi* recorded during house entry evaluations in Zungarococha village, Loreto, Peruvian Amazon

Total adult specimens collected	Specimens dissected (% Total)	Reproductive physiological status	Number by collection treatment (% of total per treatment)			*X*^2^	*P*^a^
			**Entry traps in exp. Huts (n = 598)**	**HLC inside exp. Huts (n = 522)**	**HLC inside houses (n = 452)**	5.5803	0.233
3179	1552 (48.8%)	Nulliparous (n = 433)	185 (30.7%)	128 (24.5%)	130 (28.8%)		
		Parous (n = 1,049)	387 (64.7%)	358 (68.6%)	304 (67.3%)		
		Gravid (n = 60)	26 (4.3)	16 (3.1%)	18 (4.0%)		
			**Block 1 [May–Jun] (n = 584)**	**Block 2 [Jun–Jul] (n = 474)**	**Block 3 [Jul–Aug] (n = 494)**	71.8107	< 0.0001
		Nulliparous (n = 433)	218 (37.3%)	151 (31.9%)	74 (15.0%)		
		Parous (n = 1,049)	350 (59.9%)	300 (63.3%)	399 (80.8%)		
		Gravid (n = 60)	16 (2.7%)	23 (4.9%)	21 (4.2%)		

^a^Chi Square Test (α = 0.005)

## Discussion

In this study, indoor resting patterns in wild and colonized *An. darlingi* from the Peruvian Amazon was examined and a preference for cool resting sites in both types of populations was detected. An entry unimodal peak close to midnight for wild *An. darlingi* was found, indicating that females enter houses when residents are sleeping. The *An. darlingi* resting peak (1800–2100 h) coincided with dusk when the highest temperature and lowest humidity values both inside and outside the experimental houses were recorded. No previous field or semi-field studies have reported such a resting peak for *An. darlingi*. Nevertheless, previous observations in other studies examined entry, biting, resting and exit behaviour of other Anopheles species that have a resting period outside in the vegetation after entering a house, followed by blood feeding before exiting the house. For *An. darlingi*, other studies have reported a pre-feeding resting time interval of 150 min in unsprayed conditions and 78 min in sprayed houses [68], whereas a 3–4 h indoor resting period post-feeding was found in other studies [47, 48]. It can be assumed that the females of *An. darling*i in their environment prefer to rest between (1800–2100 h) outside the houses in the vegetation waiting for the right time to enter the houses to bite later around midnight.

Moreover, *An. darlingi* resting behaviour was positively associated with temperature and was negatively associated with relative humidity both inside and outside the experimental huts. This study was carried out inside experimental huts to reduce confounding factors, which could not have been controlled using local houses. Under these conditions, *An. darlingi* preferred to rest mainly on the lower section of the door, followed by the window and roof. These locations were the closest to the points of entry, and the coolest and most humid locations inside the experimental huts, aligning with previous observations of mosquitoes preferring cooler and more humid refugia [69].

In this study, resting behaviour of *An. darlingi* was only evaluated inside experimental huts, and it was assumed that upon entering the houses, mosquitoes rested either before or after biting. However, under field conditions, *An. darlingi* may feed either inside (endophagy) or outside (exophagy) of houses. In the 1990s, this behaviour was described as variable throughout its distribution, with more exophagic behaviour in the Amazon region, while in North and Central America and Venezuela this behaviour was more endophagic [2, 19, 70]. In the late 1990s, changes in *An. darlingi* feeding were recorded with an endophagy/exophagy ratio closer to 1:1 in Central American countries and in the South American Amazon [4, 63, 64]. Recently, *An. darlingi* behavioural plasticity was reported with a possible increase in exophagy due to the use of LLINs and a reversion to endophagy once LLINs aged and lost repellency [62]. A study that examined the biting behaviour of *An. darlingi* in various communities around the city of Iquitos found higher prevalence of *Plasmodium* infection in mosquitoes collected outdoors than indoors, suggesting epidemiologically important exophagic behaviour [39]. Likewise, behavioural heterogeneity in *An. darlingi* biting behaviour on a micro geographic scale was reported, suggesting that these differences could be associated with location and collection time [40, 71]. A recent study in Brazil examined genetic diversity related to biting behaviour among *An. darlingi* mosquitoes collected in two localities of Brazil. Mosquitoes were classified according to their place of collection: inside (endophagy) and outside (exophagy), and time of collection (dusk or dawn). This was the first study to detect genetic markers associated with biting behaviour in *An. darlingi,* and the results suggested genetic differences between populations with endophagic and exophagic behaviour. However, more ecological and genomic studies are necessary to understand the genetic and environmental factors that contribute to mosquito behaviour [72].

Resting behaviour of *An. darlingi* outside homes is difficult to assess especially because the communities where females seek to feed are generally villages near rivers with abundant adjacent forest, which offers multiple potential resting sites. Although the evaluations in this study were carried out under controlled conditions, they allowed us to identify where *An. darlingi* prefer to rest after entering the houses, which was found to peak between 2200 and 2400 h based on entrance evaluations. This coincides with the peak of biting both inside and outside of homes when occupants typically carry out activities before going to sleep.

In the 1990s, *An. darlingi* was reported as predominately endophilic, which could have contributed to the success of the DDT fumigation campaigns in the 1950s [18, 19, 73]. Nevertheless, endophilic behaviour appears to have decreased due to changes in human habits and a possible behavioural adaptation of this mosquito species due to the increased insecticide use. This change from mainly endophilic to exophilic behaviour was first recorded in the 1980s and 1990s [2, 19, 38]. This same time period coincides with the last years of DDT application, and the move towards new control methodologies, including mass treatment with antimalarial drugs, and the use of new insecticides [74]. This study aimed to determine the resting and entry behaviour of female *An. darlingi* that entered a home to bite (endophagic) and rested either before or after feeding. The information obtained in this study can be useful for improving the implementation of malaria vector control programmes in this region, which rely mainly on IRS and the use of insecticide impregnated mosquito nets. Both of these methods aim to control biting mosquitoes inside houses, which tend to be open and lack window, door and eave screenings [12].

As was suggested by Sach et al*.* [23], it is important to carry out studies of indoor resting behaviour for *An. darlingi* because until now, very little has been known about the resting preferences of this species despite the direct relationship between behaviour and vector control impact. In studies carried out in Colombia in the 1980s, a tendency to rest at lower heights was observed [49], whereas in the Brazilian Amazon a tendency to rest on the ceiling was recorded [47]. The period of time that *An. darlingi* remained resting inside the house after biting ranged from a few minutes [75, 76] to two and a half hours, as recorded in the 1980s [22]. These differences in *An. darlingi* resting behaviours as well as in the other related behaviours, such as biting and entry reflect geographical and ecological variations, and likely genetic variations between populations. Additional population genetic studies are needed to better understand these differences in *An. darlingi* behaviour relevant to malaria vector control.

Despite observing similar resting patterns, a higher percentage of indoor resting was found to occur in wild mosquitoes as compared to both the lab-reared F_1_ and colonized *An. darlingi.* These differences may be due to other extrinsic or intrinsic factors, such as possible genetic differences between colonized mosquitoes reared under controlled conditions and released into semi-controlled environments such as the experimental huts. A study examining colonized *An. darlingi* did not show changes in the genetic diversity of field and F_21_ mosquitoes, suggesting that lack of genetic diversity may not have affected the colonized mosquitoes used in this study [41]. Colonized mosquitoes had a lower percentage of individuals resting, which could be due to the higher mortality of these mosquitoes since the percentages of KD in the colonized population was higher compared with the wild populations, although experiments with the F_1_ generation wild mosquitoes and colonized mosquitoes showed similar mortality. Physiological and nutritional factors may explain these differences to an extent [77, 78], yet further genetic studies between colony and wild mosquitoes are necessary to better understand these differences. Although differences in resting between wild and colonized *An. darlingi* were observed, colonized mosquitoes displayed a unimodal resting peak similar to wild mosquitoes, suggesting that colonized mosquitoes can be used in behavioural experiments during periods when wild population densities are low and collection of wild mosquitoes is limited [43, 64]. However, colonized mosquitoes originated from the same geographical area and had been reared in the laboratory for 2–2.5 years (F_26-31_), thus, repeating these experiments with colonized mosquitoes from other areas in Loreto and reared in the laboratory for a longer period of time would further confirm these suggestions.

This study showed a bimodal outdoor biting peak for *An. darlingi* (early evening at 1800 h and a second peak at 2200 h). After these initial peaks, the night-time biting activity decreased noticeably. This information was used to define the period of collection for subsequent entry evaluations. Biting activity of Anopheline species can vary geographically [2]. In studies carried out near a different study site at Puerto Almendra, which is also located within the Nanay River basin, *An. darlingi* had a unimodal outdoor landing peak that occurred between 2000 and 2100 h hours [63, 64]. Another recent study examined the biting patterns of *An. darlingi* in four communities around the city of Iquitos along the Napo and Mazan River basins and described a unimodal outdoor landing peak that occurred at 2100 h in three of the communities, while the fourth community displayed a bimodal pattern with peaks at 1900 and 2200 h [40]. Therefore, the results of this study provide further evidence that the biting activity of *An. darlingi* may vary even across nearby geographic locations.

*Anopheles darlingi* HLRs were the lowest in September 2014 and the highest in January 2015 and were associated with the rising levels of the Amazon River. These results are similar to those obtained in collections performed at Puerto Almendra in 1996 and 1997 [63, 64]. However, further analyses are necessary to determine the exact relationship between HLR patterns and other parameters such as malaria cases and rainfall patterns. Evaluations examining the entry behaviour of wild *An. darlingi* using HLCs in local houses and experimental huts as well as collection in interception traps placed in experimental huts consistently showed a unimodal trend with a peak occurring between 2200 and 2400 h, similar to findings by Sach and collaborators [23]. Peak entry just prior to midnight provides evidence that host-seeking *An. darlingi* females enter houses when inhabitants are resting and are potentially more vulnerable. The results obtained in this study are consistent with other studies conducted near Zungarococha village and located in Amazonian areas, where a single peak usually occurred before midnight [48, 63, 64, 79, 80]. The collections performed as part of this study were only conducted until midnight, but other researchers collecting near the current study location have described a secondary peak between 0200 and 0400 h [23, 81]. This bimodal behaviour is similar to that described by other researchers for other geographic areas such as southern Venezuela, Belize and areas of Brazil that are not in the Amazon region [38, 47, 82, 83].

*Anopheles darlingi* entrance activity was positively correlated with relative humidity and negatively associated with the difference of relative humidity [outdoors-indoors] (Fig. 8A). The difference between entrance rates may be related to a difference in the internal humidity of the huts between experiments (with or without interception traps) and compared to local houses. Higher humidity was recorded in local houses as compared to experimental huts during the HLCs, and the average humidity decreased even more inside the experimental huts during entry evaluations, possibly because portals were sealed when the interception traps were in place.

Given that *An. darlingi* peak indoor resting occurred between 1800 and 2100 h and the peak entry occurred between 2200 and 2400 h, it could be assumed that *An. darlingi* females can take about an hour to reach their maximum biting rate. During this time between 2100 and 2200 h it is most likely that they are probably moving from their outdoor resting places into the houses. Although the exact locations of outdoor resting sites have not been identified for *An. darlingi*, forested areas near larval habitats may serve as potential resting sites for this species. Larval habitats have historically been described as calm pools partially or well shaded, with uncontaminated water, which can be large and deep bodies of water such as ponds, lakes, pools along rivers and backwaters of streams. [8, 19, 84, 85]. Zungarococha lake, located 300 m from Zungarococha village, is adjacent to the forest and possesses a number of areas highly suitable for *An. darlingi* breeding and resting [49, 58, 66, 83]. It can be assumed that host-seeking females rest in forested areas adjacent to their larval habitats and travel 300 m towards the village after 2100 h, which is within the known flight range of female *An. darlingi* [4, 86].

Age-composition of *An. darlingi* was similar between mosquitoes collected by HLC and those collected in interception traps; the same proportion of nulliparous, parous and gravid mosquitoes was recorded within collection periods, and the same proportions were observed during entry evaluations. However, the percentage of parous females was around 60% during the first blocks of the experiments, between May to July and then began to increase by the end of the house entry evaluations that were conducted from July to August, where parous females reached 80%. This largely coincided with the number of malaria cases in the area: 73 in May; 140 in June; 152 in July; and 157 in August (Table 5). More studies should be carried out to determine the relationship between *An. darlingi* parity rate and longevity and its impact on vectoral capacity in relation to malaria incidence.

This study was carried out in Zungarococha, a malaria endemic rural area where NAMRU SOUTH has been collecting *An. darlingi* for over a decade [23, 43] Although this study was conducted during 2014–2015, and that *Anopheles darlingi* mosquitoes are capable of changing behaviour relatively quickly [62], no previous field or semi-field studies have reported on *An. darlingi* resting behaviour in the Amazon, and, furthermore, compare this behaviour between wild-type and colonized populations. Additionally, subsequent behavioural hut studies (unpublished) showed no dramatic changes in the behaviour of this malaria vector in this community.

These experimental hut studies provide valuable information on the resting and entry behaviour of *An. darlingi* from the Peruvian Amazon, in addition to providing useful information to guide indoor vector control programmes such as IRS in malaria endemic areas of the Amazon. This is also the first study showing that colonized *An. darlingi* can be used in semi-field research studies, a valuable entomological resource given the high productivity of NAMRU SOUTH’s *An. darlingi* colony year-round. These findings on *An. darlingi* resting and entrance behaviour can serve as a baseline for future trials with this vector using experimental huts to evaluate new tools for malaria control programmes.

## Data Availability

No datasets were generated or analysed during the current study.
